# Akt inhibitor deguelin aggravates inflammation and fibrosis in myocarditis

**DOI:** 10.22038/ijbms.2019.35518.8473

**Published:** 2019-11

**Authors:** Shanshan Li, Yue Wang, Chunming Zhao, Meixiang Zhang, Wei Wang, Xiaowei Yu, Jiao Huang, Zhao Wang, Bo Zhu, Chengqian Yin, Hongxing Cai

**Affiliations:** 1 Department of Forensic Medicine, Xuzhou Medical University, Xuzhou, Jiangsu 221002, China; 2 Human anatomy and Histology and Embryology, Xuzhou Medical University, Xuzhou, Jiangsu 221002, China; 3 Department of Pharmacology and Experimental Therapeutics, Boston University School of Medicine, Boston, Massachusetts 02118, USA

**Keywords:** Akt, Deguelin, Fibrosis, Inflammation, Myocarditis

## Abstract

**Objective(s):**

Myocarditis is characterized by inflammatory cell infiltration in myocardial stroma. Attenuation of tumor necrosis factor (TNF)-α and interleukin (IL)-1β is a reliable mark for improving the prognosis. Protein kinase B (Akt) plays an important role in the development and progression of myocarditis. The specific role of the natural inhibitor of Akt, Deguelin, on myocarditis has not been reported. In this study, we used deguelin to investigate the effects of natural Akt inhibitor on myocarditis in experimental autoimmune myocarditis (EAM) rats.

**Materials and Methods:**

EAM rat models were made by using Lewis rats and Deguelin was injected intraperitoneally on day 3, 6, 9, 12 and 15 after successful modeling. On day 18, rats were sacrificed and the heart weight (HW)/ body weight (BW) ratio were measured. The pathological changes, pathological scores and fibrosis area were evaluated after H.&E. and Masson’s trichrome staining. The mRNA levels of TNF-α and IL-1β were measured by RT-qPCR, while the protein expressions of TNF-α and IL-1β were detected by immunohistochemical staining and Western bolt. The protein expressions of Akt, Akt1, phosphorylated (p-) Akt and nuclear factor (NF)-κB were detected by Western bolt.

**Results:**

We found that the TNF-α and IL-1β levels, inflammatory scores and fibrosis areas were markedly increased after 18 days deguelin administration.

**Conclusion:**

Akt inhibition with deguelin may aggravate myocarditis of EAM rats.

## Introduction

Myocarditis is a cardiovascular disease can be defined as an inflammation of the myocytes (1). It is characterized by swelling, fibrosis, necrosis and inflammatory cell infiltration in connective tissue around the blood vessels. It can be delayed into dilated cardiomyopathy and heart failure, leading to sudden cardiac death (2, 3). According to reports, the sudden death caused by myocarditis in retrospective autopsy studies accounted for 34.7% of victims under 35 years old (4). The common causes of myocarditis are viral infection, bacterial infection, and autoimmune disorder (5). Histopathological features of myocarditis are myocardial interstitium with edema and inflammatory infiltration (lymphocytes and macrophages). Lymphocyte and monocyte infiltration are often observed in myocarditis, and enhanced pro-inflammatory chemokines, cytokines and circulating autoantibodies can be also detected (6). Thus, it is important to suppress inflammatory factors for improving myocarditis.

Akt is a serine/threonine-specific protein kinase that plays a key role in multiple cellular processes such as immunomodulation, proliferation, angiogenesis, migration, cell growth and metabolism (7, 8). It is involved in regulating various signaling pathways including phosphatidylinositol 3 kinase (PI3K)-Akt- mammalian target of rapamycin (mTOR), NF-κB, glycogen synthase kinase (GSK)-3β and tumor protein p53 (9-11). The PI3K-Akt-mTOR signaling pathway is one of the most extensively investigated intracellular signaling cascades involved in tumor (12), while PI3K-Akt/NF-κB/ T-cell receptor (TCR) pathway participates in immunomodulation (9). It has reported that p-Akt increased significantly upon myocarditis (13). Moreover, PI3K inhibitor LY294002 promoted apoptosis in Coxsackievirus (CVB) 3 infected cells (14), reduced myocardial damage and improved cardiac function in Experimental autoimmune myocarditis (EAM) rats by inhibiting phosphorylation of Akt (15). Akt1 is a member of the Akt family and is involved in cell growth and survival (16). Ouyang S *et al*. found Akt1-/- mice can develop ameliorated experimental autoimmune encephalomyelitis (EAE) (17). Inhibition of Akt1 aggravates the autophagic response caused by CVB3 infection in Akt1-overexpressing cells (18).These indicate that Akt played an important role in myocarditis, regulating Akt may have a therapeutic effect on myocarditis.

Deguelin, an extract of Leguminous plants, is a natural inhibitor of Akt. It exhibited anti-proliferative and anti-metastasis activities in various types of cancer (19, 20) and anti-inflammatory effect in asthmatic and cystitis (21, 22). Deguelin also improves organ survival after lung transplantation by inducing monocyte recruitment. (23). Previous study showed that deguelin inhibited p-Akt expression after myocardial infarction and then aggravated infarct size, myocardial hypertrophy, fibrosis and pathological hypertrophy (24). However, the role of deguelin in myocarditis is currently unclear. In this study, we hypothesized that Akt inhibition by deguelin plays a critical role in myocarditis. To test the hypothesis, we treated EAM rats with deguelin by intraperitoneal injection.

## Materials and Methods

***Animals and experimental protocol***

This investigation was approved by the Ethics Committee of the Xuzhou Medical University. Twenty-six male Lewis rats aged at 8 weeks (Beijing Wei Tong Li Hua Lab Animal Technology Co, Ltd) were housed in an animal facility at 22 ˚C with a 12/12 hr light/dark cycle and accessed to water and chow *ad libitum*. An EAM rat model was established according to previous report (15). Briefly, animals were immunized with purified porcine cardiac myosin (Sigma-Aldrich, M0531, St Louis, MO, USA, 10 mg/ml) mixed with equal volume of Freund’s complete adjuvant (Chondrex, 7027, Redmond, USA) in rear footpad (each 0.1 ml) on day 0. The rats in Control group received 0.01 M PBS (0.2 ml) following the same procedure. 

To testify the effect of Akt inhibition on myocarditis, deguelin (Cayman, 10010706, Ann Arbor, Michigan, USA) dissolved in dimethylsulfoxide (DMSO)/Tween-80/0.9% physiological saline (5:2:100; all Sigma) was used to treat the EAM rats by intraperitoneal injection. On day 3, day 6, day 9, day 12 and day 15 after immunization, the EAM rats were intraperitoneal injection (21) with solvent or deguelin at 1.5mg/Kg, 2 mg/Kg or 2.5 mg/Kg respectively according to previous report (25).

Body weight was measured every three days. Then the rats were sacrificed on day 18. After dissecting the heart, heart tissues were carefully harvested, washed, and weighted. Then stored those hearts at -80 ˚C for Western blot, RT-qPCR analysis or set them aside in 4% paraformaldehyde for histopathological examination. 

***Tissue preparation and histological analysis.***

Myocardial tissues were fixed in 4% paraformaldehyde for 48 hr, embedded in paraffin, cut into 4 μm thick sections, and then stained with hematoxylin-eosin or Masson’s trichrome. Pathology scores were assessed using the method previously described (26) which were calculated according to myocardial inflammatory infiltration: grade 0, no inflammation, grade 1, 1- 25% infiltration area; grade 2, 26- 50% infiltration area; grade 3, 51- 75% infiltration area and grade 4, 76-100% infiltration area. Each rat’s heart slice selects 8 views under the microscope to calculate the proportion of fibrous tissue (removing the area of perivascular collagen fibers) in each field of vision, and then the average number of fibrotic areas obtained from the 8 fields of each slice was calculated. Approximately estimate the percentage of myocardial fibrosis in a rat. Fibrillation area was measured in sections using Image-Pro^R^Plus 6.0 software (Media Cybernetics, Maryland, USA). Two pathologists assessed the histopathological scores respectively.

***Immunohistochemistry***

After deparaffinizing and hydrating these 4 μm thick sections, the Universal Two-step kit (PV-9000, Zsbio Commerce Store, Inc, Beijing, China) was used following the previously reported method (27). Primary antibodies were used as follows: rabbit-anti- interleukin (IL)-1β (1:200) from Proteintech Group (Proteintech, Wuhan, China), mouse-anti- tumor necrosis factor (TNF)-α (1:200) from Santa Cruz Biotechnology (Santa Cruz, CA, USA). Afterward, the binding sites were revealed using diaminobenzidine hydrochloride (ZLI-9018, Zsbio Commerce Store, Beijing, China). As an immunohistochemical control for the immunostaining procedure, additional sections were incubated with phosphate buffered saline (pH 7.4) in place of the primary antibody.

***Real-time quantitative PCR***

Total RNA of rat heart was extracted using Trizol reagent (15596018, Thermo Fisher, USA) and reverse translated with High- Capacity cDNA Reverse Transcription Kit (4368814, Thermo Fisher, USA) following the manufacturer’s protocol. Real-time PCR was performed using the 7500 Real- Time PCR Products (Applied Biosystems) with PowerUpTM SYBR^R^ Green Master Mix (A25742, Thermo Fisher, USA). The reaction program was as follows: 50 ˚C for 2 min, 95 ˚C for 2 min, following by 40 cycles of 95 ˚C for 15 min, 57 ˚C for 15 min, 72 ˚C for 1 min. Copy numbers of the GAPDH housekeeping gene (B661204- 0001, BBI Life Sciences), TNF-α and IL-1β were determined. The following primers were designed and synthesized by GenScript Biotech Corp (Nan Jing, China): TNF-α, forward 5’-GCATGATCCGAGATGTGGAA-3’, reverse 5’- TGAGAAGAGGCTGAGGCACA-3’(98bp); IL-1β, forward 5’-AAATGCCTCGTGCTGTCTGA-3’, reverse 5’- TTGGGATCCACACTCTCCAG-3’ (218 bp). The relative expression levels of the genes were performed using the comparative quantification cycle (2^-^^ΔΔCT^) method (21). Each sample was analyzed in triplicate.

***Protein preparation and immunoblotting assay*** **.**

The protocols of Western blotting were based on a previously reported method (28, 29). Each 100mg heart sample was homogenized in 0.99 ml RAPI lysis buffer (P0013B, Beyotime Institute of Biotechnology, Shanghai, China) supplemented with 10 μl PMSF (ST506, Beyotime Institute of Biotechnology, Shanghai, China) and 10 μl/1ml phosphatase inhibitors (KGP602, KeyGEN BioTECH, Jiangsu, China). The mixture was allowed to stand on ice for 30 min. Then, centrifuged at 12,000×g for 15 min at 4 ˚C, collected supernatant. Protein concentrations were measured with the Enhanced BCA Protein Assay Kit (P0010, Beyotime Biotechnology, Shanghai, China). Twenty μg of total protein was separated by 10% sodium dodecyl sulphate-polyacrylamide gel electrophoresis (SDS-PAGE), transferred to a polyvinylidene difluorid (PVDF) membrane (ISEQ00010, Merck Millipore Ltd, Darmstadt, Germany). After blocking with 5% non-fat milk, PVDF membrane was incubated with primary antibody overnight at 4 ˚C. The primary antibodies were used as follows: rabbit-anti-GAPDH (1:700, 60004), mouse-anti-Akt (1:2000, 60203-2-lg) and rabbit-anti-IL-1β (1:800, 16806) from Proteintech Group (Proteintech, Wuhan, China); rabbit-anti-p-Akt (ser473, 1:800, AF3263) and mouse-anti-NF-κB p65 (1:900, sc-8008), mouse-anti-Akt1 (1:700, sc-5298) and mouse-anti-TNF-α (1:600, sc-12744) from Santa Cruz Biotechnology (Santa Cruz, CA, USA). Goat Anti-Rabbit IgG (D110058, Sangon Biotech, Shanghai, China) and Goat Anti-Mouse IgG (H+L) (VA002, VICMED, Jiangsu, China) were used as secondary antibodies. Then the PVDF membranes were chemiluminescence with Beyozol ECL Plus (P0018, Beyotime Institute of Biotechnology, Shanghai, China) by Tanon-5200Mult chemiluminescence imaging analysis system (Tanon, Shanghai, China). The bands of the blot were quantified by densitometry using Image-Pro^R^ Plus software (Media Cybernetics, Maryland, USA).

***Statistical analysis***

All data were expressed as means ± standard deviation (SD) and statistical analysis was performed by SPSS 17.0 (SPSS Inc., Chicago, IL, USA). The statistical significance of differences was determined using One-way ANOVA for multiple comparisons with LSD-t for analysis between groups. Values of *P*<0.05 were considered statistically signiﬁcant. Graphs were created by Prism 7.00 (GraphPad Software Inc., San Diego, CA, USA).

## Results

***EAM rat dead soon after high doses of deguelin injection***

**Figure 1:**
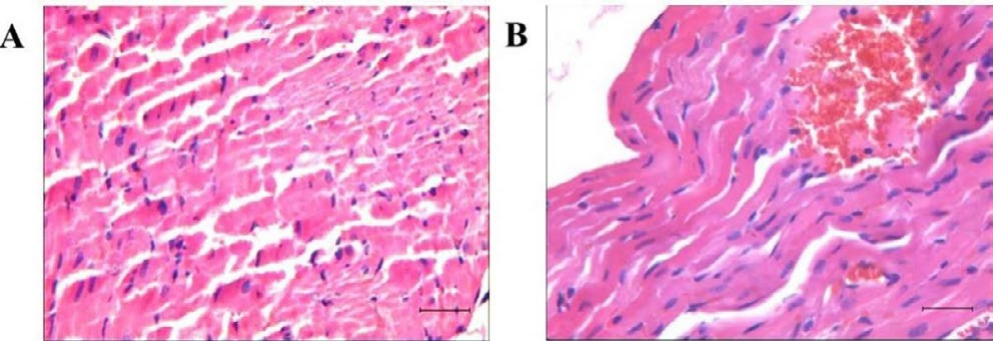
Morphological analysis of the myocardium in high dose deguelin groups. A) Extensive fragmental myocardium. B) Focal myocardial hemorrhage. The bar represented 100 μm

To evaluate the effect of deguelin, we treated EAM rats with 1.5, 2 or 2.5 mg/Kg deguelin respectively. We found that all rats in control and EAM+Vehicle group survived at day 18, while rats in EAM+1.5mg/Kg Deguelin group survived 3/4. However, all rats in EAM+2 mg/Kg and 2.5 mg/Kg Deguelin group died soon after the first deguelin injection (Table 1). We performed H&E staining on the heart of dead rats to find out the cause of death. We found multi-focal myocardial hemorrhage as well as extensive fragmental myocardium and wave-like changes (Figure 1). It suggested that high dose deguelin had acute toxicity on EAM rat.

***Effect of deguelin on heart weight/body weight (HW/BW)***

We recorded the changes of rat’s BW in each group (Table 2). We found that the BW of rats in EAM+Vehicle (abbreviated as EAM) group and EAM+1.5 mg/Kg deguelin (abbreviated as EAM+Deguelin) group were obvious lower compared with the control group (*P*< 0.01). However, there was no significant difference between the body weight in EAM group and EAM+Deguelin group on the same day. The remaining rats were sacrificed at day 18 and their heart weights were measured. The heart weight in EAM group and deguelin group rats were markedly increased. As a result, the HW/BW ratio in EAM group and EAM+Deguelin group were obviously increased compared with the control group (Table 2) (*P*<0.01). However, the HW/BW ratio had no significant differences between EAM+Deguelin group and EAM group. It suggested that deguelin had no obvious effect on HW& BW of EAM rats.

***Deguelin inhibited Akt phosphorylation***

**Figure 2:**
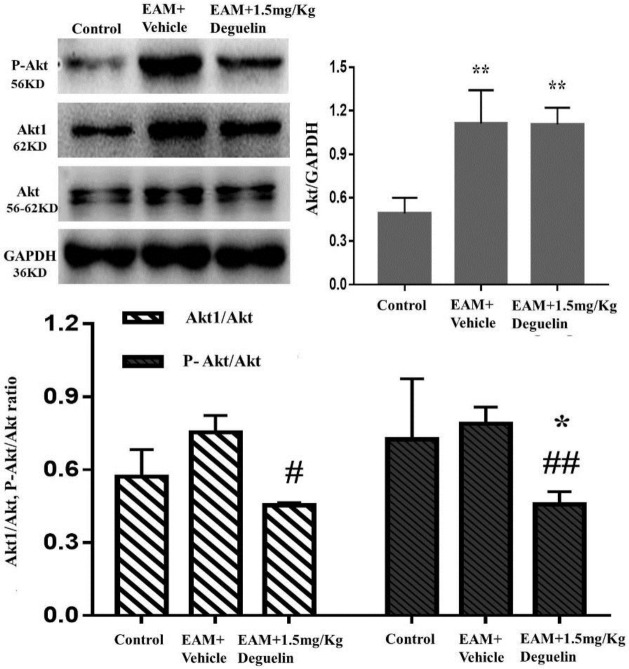
Deguelin inhibited Akt phosphorylation. Selected picture of Akt, Akt1 and p-Akt protein levels in each experimental group. The expression of Akt1 and p-Akt in EAM+1.5 mg/Kg Deguelin group was lower than that in the EAM+Vehicle group. *P<0.05 or **P<0.01 vs Control group; #*P*< 0.05 or ## *P*<0.01 vs EAM+Vehicle group

Western blotting revealed that the total Akt, Akt1 and p-Akt in EAM group increased compared with the control group. Furthermore, we observed a clear reduction of Akt1 and p-Akt levels, whereas total Akt levels remained unchanged in deguelin group compared with EAM group (Figure 2). The result confirmed that deguelin decreased Akt1 and inhibited Akt phosphorylation.

***Deguelin aggravated myocarditis***

**Figure 3:**
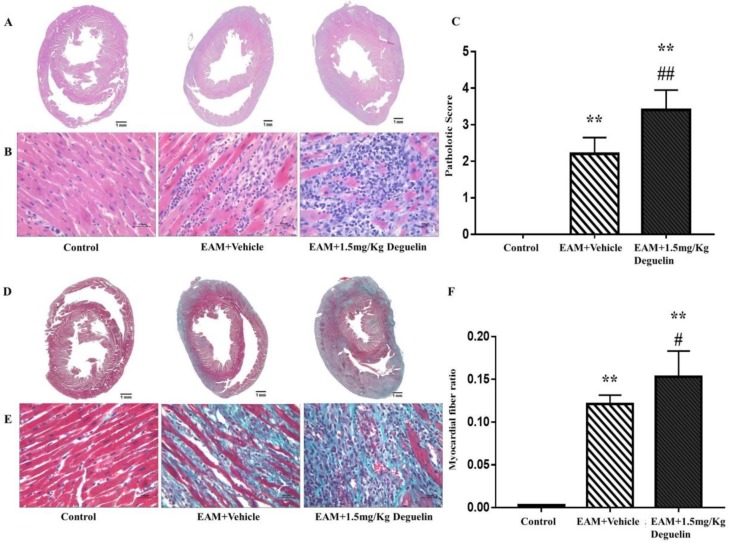
Morphological changes after deguelin administration. A & B) HE-stained sections of the Control group, EAM+Veh group and EAM+1.5 mg/Kg Deg group. C) Pathological scores of each group (Two pathologists scored the sections by double-blind reading.). D & E) Masson stained sections of the Control group, EAM+Vehicle group and EAM+1.5 mg/Kg Deg group. F) The ratio of myocardial fibrosis area to total myocardial area. **P<0.01 vs. Control group; #*P*<0.05 or ##*P*<0.01 vs. EAM+Vehicle group. The bar represented 100 μm

To measure the inflammation and fibrosis degree, we performed the cardiac cross section on each group. We found that myocardial fibers were intact and well-arranged without inflammatory cell infiltration in the control group. Tissues from EAM group revealed multifocal inflammatory infiltration in stroma with a few myocardial necrosis and collagen deposition. Tissues from EAM+Deguelin group showed extensive inflammatory exudation with severe cardiomyocyte necrosis and collagen deposition (Figure 3). We calculated the pathology scores of each section, which was much higher in EAM+Deguelin group than in the EAM group (Figure 3C) (*P*<0.01). Moreover, the area of myocardial fibrosis in the EAM+Deguelin group was significantly higher than that in the EAM group (Figure 3F) (*P*<0.05). In brief, EAM+Deguelin aggravated the inflammatory infiltration and myocardial fibrosis of EAM rats.

**Figure 4:**
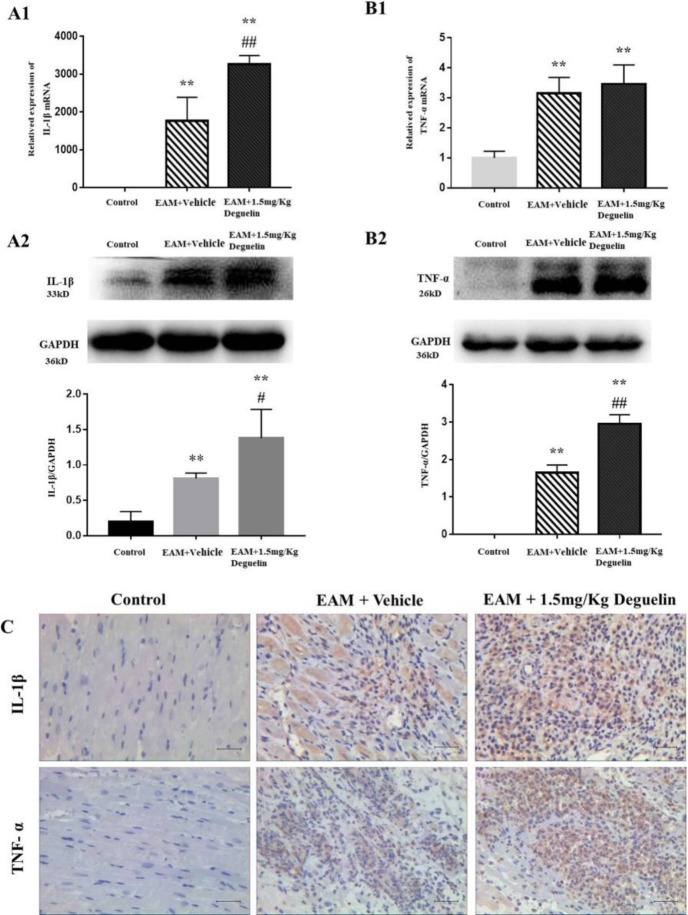
Deguelin increased the expression of IL-1β and TNF-α. Detection of IL-1β and TNF-α by RT-qPCR (A1 & B1) and Western blot (A2 & B2). **P<0.01 vs. Control group; #*P*<0.05 or##*P*<0.01 VS. EAM+Vehicle group. C Immunohistochemical analysis of IL-1β and TNF-α. The bar represented 100 μm

**Figure 5:**
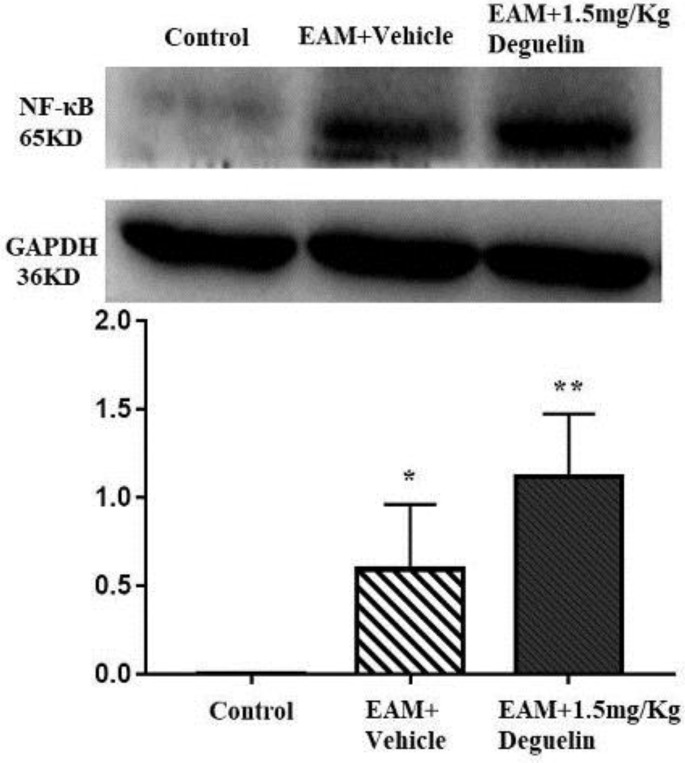
Deguelin promoted the expression of NF-κB protein expression. *P<0.05 or **P<0.01 vs. Control group

We tested inflammatory cytokines by using RT-qPCR, Western blot and immunohistochemical staining. RT-qPCR showed that rats in EAM group had a higher level of IL-1β than in the control group (*P*<0.01), and in EAM+Deguelin group had even higher (*P*<0.01) (Figure 4A1). Compared with the control group, the TNF-α expression in the EAM group was significantly increased (*P*<0.01) (Figure 4B1). Similarly, the EAM+Deguelin group had higher TNF-α expression values than the control group (*P*<0.01) (Figure 4B1). Western blotting showed that IL-1β and TNF-α in deguelin group increased significantly compared with the EAM group (*P*<0.05, Figure 4A2) (*P*<0.01, Figure 4B2). Immunohistochemical staining confirmed the results above. IL-1β and TNF-α were increased in infiltrating mononuclear cells of treated myosin-immunized rats. Among them, the number of IL-1β and TNF-α monocytes expressed in the EAM+Deguelin administration group was higher than that in the EAM group (Figure 4C). We also tested the expression of protein NF-κB, it was highly expressed in EAM group (*P*<0.05) and even higher in EAM+Deguelin group (*P*<0.01) (Figure 5).

## Discussion

The present study investigated the effects of chronic pharmacological blockade of Akt phosphorylation by deguelin in EAM rats’ model. We found that deguelin promoted inflammation and fibrosis of myocardium, increased the expression of proinflammatory factors, aggravated myocarditis.

EAM rat in our experiment was immunized with porcine cardiac myosin together with complete Freund’s adjuvant. Histopathology confirms that myocarditis is a T cell-mediated immune response, mainly infiltrating myocardium by CD11b+ and CD4+ T cells (30). EAM is a CD4+ T cell-mediated autoimmune disease. The original CD4+ T cells can generally differentiate into two different cell subpopulations (Th1 and Th2) and the acute stage of EAM is vitally interrelated to Th l CD4+ T cell-mediated immune response (31). EAM is characterized by severe myocardial damage and inflammatory cell infiltration, which mimics “human fulminant myocarditis in the acute phase and dilated cardiomyopathy (DCM) in the chronic phase” (32). Therefore, EAM rat is commonly used as a model to study acute and chronic myocarditis and dilated cardiomyopathy (33-35).

Studies have shown that inflammatory factors such as TNF-α and IL-1β play key roles in the induction and progression of EAM (36). Their levels are positively correlated with the severity of the myocarditis (37). TNF-α promotes EAM development and cardiomyocyte apoptosis, reduces myocardial contractility by binding its low molecular weight receptor p55 (38). TNF-α interferes with cardiac contractile function by reducing L-type voltage-dependent Ca^2+^ channels in cardiomyocytes (39) IL-1β, as well, reduces cardiomyocyte contractility and destroys Ca^2+ ^homeostasis in cardiomyocytes, leading to spontaneous arrhythmias (40). NF-κB also plays an important role in regulating the induction of a variety of pro-inflammatory cytokines in inflammation. NF-κB activation is a hallmark of chronic inflammatory diseases (41). Inhibition of NF-κB activity can block the production of pro-inflammatory cytokines in myocardial tissue, thereby preventing the development of myocarditis (42). Known inducers of NF-κB activity are highly variable including reactive oxygen species (ROS), TNF-α and IL-1β (41). Moreover, NF-κB activation can induce gene expression of the pro-inflammatory cytokines IL-1β and TNF-α (43). Our study showed that the expressions of IL-1β, TNF-α and NF-κB were significantly up-regulated after deguelin administration. Moreover, the pathology score and fibrosis area increased in deguelin group. These results indicated that deguelin promoted the inflammation of EAM rats. 

Deguelin is a natural inhibitor of Akt (11). Akt pathway is a central regulatory factor in many cardiovascular pathological processes such as cardiac hypertrophy, atherosclerosis and vascular remodeling (8). Studies have shown that Akt is activated in myocarditis and Akt network related genes markedly changed during murine acute chagasic myocarditis according to microarray analysis (44, 45). He *et al*. reported salidroside mitigated sepsis-induced myocarditis by downregulating PI3K/Akt/GSK-3β signaling (46); Song *et al*. found curcumin protected mice from coxsackievirus B3-induced myocarditis by inhibiting the PI3K/Akt/NF-κB pathway (13); Other reported PI3K inhibitor LY294002 promoted apoptosis in coxsackievirus 3 infected HeLa cells and reduced cardiac necrosis, inflammatory infiltrates, and CD3(+) T cells in EAM mice via down-regulation of p-Akt (14,15). These suggest that inhibiting PI3K/Akt pathway had potent effects against myocarditis. However, our study showed a different result. We found that deguelin could efficiently decrease the expression of Akt1 and p-Akt but not Akt, Besides, deguelin increased the IL-1β, TNF-α expressions, aggravated the inflammation and fibrosis, therefore, deteriorated myocarditis. There may be some reasons for the contradiction: first, deguelin inhibits Akt by both PI3K dependent and independent pathways. On one hand, deguelin inhibits PI3K activity and reduced p-Akt levels and activity (47). On another hand, deguelin induces apoptosis in head and neck squamous cell cancer cell lines by targeting both EGFR-Akt and IGF1R-Akt pathways (48), which were related to TNF-α and NF-κB (49). Second, deguelin may regulate the phenotype through TNF-NF-κB pathway (50). Moreover, Li *et al*. reported that down-regulation of microRNA-146b can inhibit Akt phosphorylation and promote NF-κB expression, thereby aggravating the occurrence of vascular inflammation in myocardial infarction (51).

**Table 1:** Survival number of rats in each group

	**Control**	**E** **AM** **+Vehicle**	**EAM+** **Deguelin**		
			**1** **.5 m** **g/Kg**	**2** ** m** **g/Kg**	**2** **.5 m** **g/Kg**
Survival n.	5	5	6	0	0
Dead n.	0	0	2	4	4
Total n.	5	5	8	4	4

EAM: Experimental autoimmune myocarditis

**Table 2:** Heart weight (HW), body weight (BW) and heart weight/body weight (HW/BW) ratio in each group

**Day**	**Control** **（BW）**	**EAM+vehicle** **（** **BW** **）**	**EAM+Deguelin（BW）**		
			**1.5 mg/Kg **	**2 mg/Kg**	**2.5 mg/Kg**
0	236.62±27.93	233.46±18.35	233.10±5.91	219.75±8.77	231.75±2.06
3	262.14±19.98	223.82±13.85^**^	219.50±4.68^**^	197.75±9.79^**^	210.95±2.59^**^
6	274.82±22.70	234.02±13.75^**^	230.14±12.49^**^	-	-
9	284.92±21.51	244.84±14.11^**^	248.14±9.58^**^	-	-
12	303.54±22.27	240.04±19.09^**^	242.74±17.27^**^	-	-
15	315.98±21.43	227.90±17.16^**^	223.82±7.25^**^	-	-
18	330.96±20.92	226.48±25.18^**^	233.00±7.17^**^	-	-

“**”*P*< 0.01 vs. Control on the same day

EAM: Experimental autoimmune myocarditis

EAM: Experimental autoimmune myocarditis

EAM: Experimental autoimmune myocarditis

EAM: Experimental autoimmune myocarditis

Our results are consistent with Buss *et al*’s study which showed that chronic blockade of Akt by deguelin increased cardiomyocyte sizes and heart weights, deteriorated cardiac remodeling and function in a rat myocardial infarction model (24). Buss *et al*. and us both used deguelin. Previous reports show that deguelin promoted apoptosis, suppressed the adhesion, migration and invasion of cancer cells (19, 20), which indicated an anticancer effect of deguelin. It also exhibited a cardiotoxic effect. We observed acute myocardial damage: multi-focal myocardial hemorrhage, extensive fragmental myocardium and wave-like changes in the hearts of dead rats treated with deguelin. While Buss *et al*. reported that deguelin did not deteriorate the heart function of sedentary rats and no normal animals died after deguelin treatment (24). These indicated that deguelin might have no negative effect on the healthy heart, meanwhile, it deteriorated cardiac function when the heart was injured. However, most of the research on deguelin is now on the cell level, and there are few studies on biological individuals. The effect of deguelin administration on myocarditis has not been reported. As an anticancer drug with strong biological activity, the deguelin is commonly used in the research of tumor therapy (19, 52). According to the results of this study, it is necessary to study its anti-tumor activity while also considering its side effects.

## Conclusion

Our study found that inhibiting Akt phosphorylation by deguelin aggravates myocarditis in EAM rat model. We should pay attention to the patients with cardiovascular diseases when using Akt inhibitor-deguelin.

## Conflict of Interest

The authors have no conflicts of interest.
